# Oligometastatic state predicts a favorable outcome for renal cell carcinoma patients with bone metastasis under the treatment of sunitinib

**DOI:** 10.18632/oncotarget.8568

**Published:** 2016-04-04

**Authors:** Xiaolin Lu, Weijie Gu, Hailiang Zhang, Yao Zhu, Guohai Shi, Dingwei Ye

**Affiliations:** ^1^ Department of Urology, Fudan University Shanghai Cancer Center, Shanghai, People's Republic of China; ^2^ Department of Oncology, Shanghai Medical College, Fudan University, Shanghai, People's Republic of China

**Keywords:** renal cell carcinoma, bone metastasis, oligometastasis, prognosis

## Abstract

**Background:**

The aim of the study was to investigate whether RCC patients with oligometastatic state of bone metastasis treated with sunitinib had a favorable clinical outcome.

**Results:**

22 patients were classified into oligometastatic state of bone metastasis with a median OS of 30.1 months (95%CI: 26.3 to 33.8 months). The 45 patients with non-oligometastatic state had a median OS of 12.7 months (95%CI: 9.43 to 16.0 months). Kaplan-Meier analysis showed significant difference between them (Log Rank test p<0.001). When we set patients with only multiple bone (at least 5 sites) metastases as a single group, there was still significant difference between oligometastatic state group and non-oligometastatic state groups. In multivariate Cox proportion hazard ratio analysis, metastatic states (p=0.012), MSKCC score (p=0.002), ECOG (p=0.001) and lymph nodes metastasis (p=0.000) were significantly associated with prognosis. The integration of metastatic state into the MSKCC risk model improved the c-index from 0.651 to 0.752

**Method:**

67 patients from Fudan University Shanghai Cancer Center with bone metastatic RCC were divided into 2 metastatic states. One included those with oligometastatic state of bone metastasis with less than 5 sites of bone metastasis. The other involved those patients with multiple bone metastases (at least 5 sites) or together with other sites of metastasis. Then patients with only multiple bone (at least 5 sites) metastases were set into a single group.

**Conclusion:**

RCC patients with oligometastatic state of bone metastasis treated with sunitinib had a favorable clinical outcome.

## INTRODUCTION

Renal cell carcinoma takes about 3% of all malignancies in adult, with 61560 new cases of renal cancer and 14080 deaths of the disease in 2015 according to the statistics of the United States [1]. Although certain development has been achieved in diagnosis and treatment strategies of RCC, at diagnosis one third of the patients present metastatic disease and 20–40% of RCC patients will develop metastasis even after nephrectomy [2].

Lung is the most common site of metastasis in RCC patients, affecting about 45–50% of patients with metastatic disease [3]. Skeleton and liver come with second and third place with involvement of 30% and 20%, respectively [3]. Previous studies have suggested that the presence of bone metastasis (BMs) is correlated with poor prognosis with a median survival of 12 months [4, 5]. BMs in RCC is usually a lytic progress causing potential morbidity because of skeletal related events (SREs), defined as a pathological fracture, spinal cord compression or hypercalcemia which needs surgical intervention or requirement for palliative radiotherapy to bone [6].

However, long survival in patients with BMs from RCC is not a rare event [7], especially after the introduction of tyrosine kinase (TKIs) and mTOR inhibitors which has completely revolutionized the therapeutic scenario of mRCC. Sunitinib is an oral tyrosine kinase inhibitor (TKI) targeting the vascular endothelial growth factor receptor (VEGFR) and the platelet-derived growth factor receptor (PDGFR). Its effect was proved by a phase III clinical trial comparing mRCC patients under the treatment of INF-α or sunitinib. The result indicated that sunitinib could significantly improve progression-free survival (PFS) compared with interferon-α (IFN-α) (11 vs. 5 months), as well as overall survival (OS) (26.4 vs 21.8 months).

The oligometastatic state is usually defined as the presence of five or fewer metastatic or recurrent lesions in a case of solid malignancy [8]. Studies have shown that oligometastatic state is a significant favorable factor in tumors like lung and prostate cancer if treated with aggressive therapy [9].

The aim of the study was to investigate whether RCC patients with oligometastatic state of bone metastasis treated with sunitinib had a favorable clinical outcome.

## PATIENTS AND METHODS

The study was approved by Ethical Committee of Fudan University Shanghai Cancer Center, and written informed consent was obtained from all patients. 245 patients with advanced RCC treated with sunitinib as first-line therapy from Fudan University Shanghai Cancer Center (FUSCC) were retrospectively reviewed. Among them 67 developed bone metastasis. Bone metastatic sites were screened by Emission Computed Tomography (ECT) or positron emission tomography *(PET) confirmed by one experienced radiologist through* total body contrast-enhanced CT or MRI. All of these 67 patients were divided into 2 metastatic states. One included those with oligometastatic state of bone metastasis which means these patients only had less than 5 sites of bone metastasis. The other involved those patients with multiple bone metastases (at least 5 sites) or together with other sites of metastasis. Furthermore, the patients with non-oligometastatic state were divided into two groups. One included those with only multiple bone (at least 5 sites) metastases and the other involved those with other sites of metastases.

Clinicopathological characteristics, including age, gender, metastatic sites, LDH level, calcium level, hemoglobin, disease free interval (DFI), skeletal related events (SREs), eastern cooperative oncology group performance status (ECOGPS) and Memorial Sloan Kettering Cancer Center score (MSKCC) were obtained from electronic records (Table 1). Patients were regularly followed up by telephone or in the clinic once every 3 months. Events, such as tumor recurrence, progression, metastasis and death, were recorded.

**Table 1:** Baseline clinical characteristics of the RCC patients with bone metastasis treated with sunitinib

Variables	Number (percentage)
Gender	
male	51(76.1%)
female	16(33.9%)
Age (Years)	
≥58	37(55.2%)
<58	30(44.8%)
MSKCC score	
Low risk	33(49.3%)
Intermediate risk	31(46.3%)
High risk	3(4.4%)
KPS	
>70	64(95.5%)
≤70	3(4.5%)
ECOG	
0	47(70.1%)
1	16(23.9%)
2	1(1.5%)
3	2(3.0%)
DFI	
≥1y	35(52.2%)
<1y	32(7.8%)
Hemoglobin	
High	54(80.6%)
Low	13(19.4%)
Corrected calcium	
≤10 mg/dL	65(97.0%)
>10 mg/dL	2(3.0%)
LDH	
High	6(9.0%)
Low	61(91.0%)
Skeletal related events	
YES	45(67.2%)
No	22(32.8%)
Metastasis other than bone	
Lung	42
Liver	8
Lymph nodes	13
Brain	2
Others	10

Abbreviations: MSKCC=Memorial Sloan Kettering Cancer Center: ECOG=Eastern Cooperative Oncology Group; KPS= Karnofsky Performance Status; DFI = disease-free interval; LDH = lactate dehydrogenase.

### Statistical analysis

Overall survival was calculated from the date of diagnosis to the date of death or last follow-up. Disease free interval was defined as the time from nephroectomy to disease recurrence or metastasis. Patients without events or death were recorded as censored at the time of last follow-up. Spss software was used to perform statistical analysis. Differences in the distribution of variables between oligometastatic state and non-oligometastatic state were evaluated using the chi-square test (Table 2). Survival curves were constructed using the Kaplan–Meier method, with log-rank tests used to assess the differences between the groups. Adjusted hazard ratio (HR) with 95% confidence intervals (95% CIs) was calculated using Cox proportional hazards models. Harrell's c-index was used to evaluate the predictive accuracy of Cox proportional hazards models which is analogous to the area under the receiver operating characteristic curve for censored data [10]. A two-sided P-value <0.05 was considered to indicate statistical significance.

**Table 2:** Differences in the distribution of variables between oligometastatic state and non-oligometastatic state

Variables	Oligometastasis		p value
	yes	no	
All patients	22(32.8%)	45(67.2%)	
Gender			
male	18(81.8%)	33(73.3%)	0.757
female	4(18.2)	11(26.7)	
Age (Years)			
≥58	10(45.5%)	27(60.0%)	0.303
<58	12(54.5%)	18(40.0%)	
MSKCC score			
Low risk	13(59.1%)	20(44.4%)	0.141
Intermediate risk	8(36.4%)	23(51.1%)	
High risk	1(4.5%)	2(4.5%)	
ECOG			
0	15(68.2%)	32(71.2%)	0.568
1	7(31.8%)	10(22.2%)	
2	0	1(2.2%)	
3	0	2(4.4%)	
DFI			
≥1y	13(59.1%)	22(48.9%)	0.45
<1y	9(40.9%)	23(51.1%)	
Hemoglobin			
High	17(77.3%)	37(82.2%)	0.745
Low	5(22.7%)	8(17.8%)	
Corrected calcium			
≤10 mg/dL	22(100%)	43(95.6%)	1
>10 mg/dL	0(0%)	2(4.4%)	
LDH			
High	0(0%)	6(13.3%)	0.167
Low	22(100%)	39(86.7%)	
Skeletal related events			
YES	9(40.9%)	37(82.2%)	0.002
No	13(59.1%)	8(17.8%)	
Dose reduction			
Yes	12(54.5%)	13(28.9%)	0.06
No	10(45.5%)	32(71.1%)	

Abbreviations: MSKCC=Memorial Sloan Kettering Cancer Center; ECOG=Eastern Cooperative Oncology Group; DFI = disease-free interval; LDH = lactate dehydrogenase

## RESULTS

### Patients characteristics

We retrospectively reviewed clinical data of 67 patients with RCC BMs treated with sunitinib (50 mg/day; 4 weeks on and 2 weeks off) followed from May 2008 to June 2015. All the patients went through nephrectomy before the use of sunitinib. Median age of the patients was 58 years old. Among them 51 (76.1%) were male; 59 (88.1%) were clear cell type, while 8 (11.9%) presented with other histology; 45 patients had at least one SRE during the disease course, the median number of which was 1 ranging from 0 to 4; 3 were treated with sorafenib before the use of sunitinib; 25 patients reduced the dose of sunitinib to 37.5 mg/day due to adverse events. The median overall survival (OS) of these patients was 13.6 months.

### Oligometastatic state of bone metastasis predicted a favorable outcome for renal cell carcinoma patients under the treatment of sunitinib

**Figure 1:**
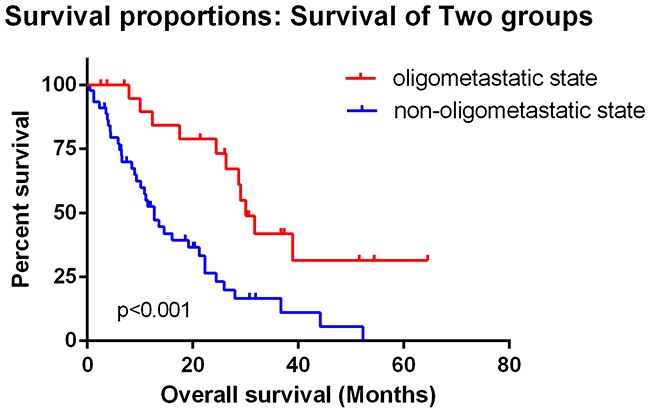
Kaplan–Meier plots of overall survival showed significant difference between oligometastatic state and non-oligometastatic state (Log Rank test p<0.001) which means oligometastatic state of bone metastasis was a favorable outcome for renal cell carcinoma patients

**Figure 2:**
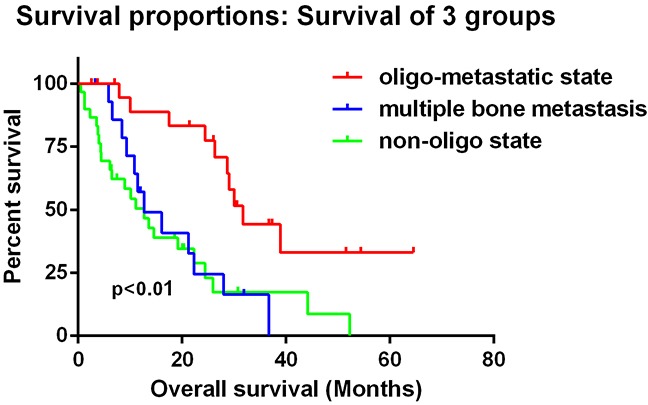
When we set patients with only multiple bone (at least 5 sites) metastases as a single group, there was still significant difference between oligometastatic state group and non-oligometastatic state groups, but there was no difference between multiple bone metastases group and non-oligo group

All of these 67 patients were divided into 2 states. 22 patients were classified into oligometastatic state of bone metastasis with a median OS of 30.1 months (95%CI: 26.3 to 33.8 months). The 45 patients with non-oligometastatic state had a median OS of 12.7 months (95%CI: 9.43 to 16.0 months). Among non-oligometastatic state group, 15 patients had only bone metastases with a median OS of 12.7 months (95%CI: 5.15 to 20.3 months) and 30 had both bone metastases and other sites of lesions with a median OS of 12.7 months (95%CI: 7.15 to 18.3 months). Kaplan-Meier analysis showed significant difference between oligometastatic state and non-oligometastatic state (Log Rank test p<0.001) which means oligometastatic state of bone metastases was a favorable outcome for renal cell carcinoma patients (Figure 1). When we set patients with only multiple bone (at least 5 sites) metastases as a single group, there was still significant difference between oligometastatic state group and non-oligometastatic state groups, but there was no difference between multiple bone metastases group and non-oligo group (Figure 2).

### Relative factors that affect overall survival of the patients with bone metastasis under the treatment of sunitinib

In univariate Cox proportion hazard ratio analysis, higher LDH (HR: 4.208, p=0.005), MSKCC score (HR:2.25, p=0.006), ECOG (HR:2.079, p=0.001), lymph nodes metastasis (HR: 2.094, p=0.036), lung metastasis (HR: 2.560, P=0.003) and metastatic state (HR:3.201, p=0.001) were significantly correlated with prognosis. A reduced model was used in multivariate cox proportion hazard ratio analysis. Variables that were significant in univariate analysis were included in the multivariate analysis, the results of which indicated that MSKCC score (HR:2.73, p=0.002), ECOG (HR:2.279, p=0.001), lymph nodes metastasis (HR: 4.463, p=0.000) and metastatic state (HR:3.468, p=0.012) were correlated with overall survival.(Table 3)

**Table 3:** Univariate and multivariate analyses to predict overall survival

Variables	Univariate		Multivariate	
	HR (95%CI)	P value	HR (95%CI)	P value
Gender				
male	0.965 (0.497-1.872)	0.916	/	/
female				
Age (Years)	1.01 (0.976-1.045)	0.563	/	/
MSKCC score				
Low risk	2.25 (1.260-4.018)	0.006	2.73 (1.425-5.229)	0.002
Intermediate risk				
High risk				
ECOG				
0	2.079 (1.278-3.384)	0.003	2.279 (1.397-3.717)	0.001
1				
2				
3				
KPS				
>70	15.042(3.73-60.60)	0		
≤70				
DFI				
≥1y	1.406 (0.784-2.522)	0.253	/	/
<1y				
Hemoglobin				
High	1.001 (0.481-2.084)	0.997	/	/
Low				
Corrected calcium				
≤10 mg/dL	0.049	0.868	/	/
>10 mg/dL				
LDH				
High	4.208 (1.552-11.409)	0.005	/	/
Low				
Metastasis other than bone				
Lung	2.56 (1.387-4.724)	0.003	1.585 (0.707-3.552)	0.264
Liver	2.092 (0.852-5.135)	0.107	/	/
Lymph nodes	2.094 (1.050-4.173)	0.036	4.463 (2.023-9.844)	0
Brain	1.451 (0.346-6.074)	0.611	/	/
Others	1.481 (0.581-3.778)	0.411	/	/
Number of SREs	1.273 (0.670-2.421)	0.461		
Metastatic state				
oligometastasis	3.201 (1.601-6.402)	0.001	3.468 (1.316-9.141)	0.012
non-oligometastasis				

Abbreviations: MSKCC=Memorial Sloan Kettering Cancer Center; ECOG=Eastern Cooperative Oncology Group; KPS= Karnofsky Performance Status;DFI=disease-free interval; LDH=lactate dehydrogenase; HR=hazards ratio; CI=Confidence interval; SREs=Skeletal related events

### Incorporation of metastatic state into the MSKCC risk model

The integration of metastatic state into the MSKCC risk model improved the c-index from 0.651 to 0.752 (Table 4). Multivariable analysis of predictors of OS from the diagnosis of bone metastasis in patients with RCC indicated ECOG (p=0.019), lymph-node (p = 0.036) and lung (p = 0.004) metastasis were independent prognostic factors for OS.

**Table 4:** Incorporation of oligometastasis into the MSKCC risk model

	Model 1	P value*	Model 2	P value†
**Variables**	KPS≤70	0.002	KPS≤70	0.003
	DFI <1 year	0.237	DFI <1 year	0.263
	Hemoglobin < LLN	0.718	Hemoglobin < LLN	0.791
	Corrected calcium>10mg/dL	0.981	Corrected calcium>10mg/dL	0.980
	LDH >1.5 × ULN	0.057	LDH >1.5 × ULN	0.405
			Metastatic state	0.002
**C-index** **(95%CI)**	0.651 (0.571-0.732)		0.752 (0.676-0.829)	

Abbreviations: MSKCC=Memorial Sloan Kettering Cancer Center; KPS= Karnofsky Performance Status; DFI = disease-free interval; LDH = lactate dehydrogenase;LLN = lower limit of normal

* calculated in the multivariate analysis, which includes ECOG, DFI <1 year, hemoglobin<LLN, corrected calcium >10mg/dL, and LDH >1.5 × ULN as variables.

† calculated in the multivariate analysis, which includes ECOG, DFI <1 year, hemoglobin<LLN, corrected calcium >10mg/dL, LDH >1.5 × ULN, and the metastatic state as variables.

## DISCUSSION

In this study, we reviewed 67 patients with RCC BMs treated with sunitinib. We believe that patients with bone metastasis are not necessarily associated with poor overall survival. Oligometastatic state predicts a favorable outcome for renal cell carcinoma patients with bone metastasis.

Cancer with metastasis has been considered as an end-stage disease, the treatment of which is systemic management, including chemotherapy, target therapy or hormone therapy [9]. Usually, these patients have poor clinical outcomes. However, a proportion of patients with fewer sites of metastasis turn out to live longer which gave birth to the definition of oligometastatic state. The concept of an oligometastatic state was first introduced by Hellman et al. It is defined as an intermediate state (≤5 metastases) between limited primary and polymetastatic cancers [11, 12]. These patients, if treated with aggressive therapy may have a satisfactory survival even comparable to non-metastatic disease [9]. The biological basis of the oligometastatic phenotype is yet to be discovered. In renal cell carcinoma, Wuttig et al. identified 135 genes that were differentially expressed between ‘few’ (<8) or ‘many’ (>16) pulmonary metastases and found non-oligometastatic tumors were enriched by genes that regulate the cell cycle [13].

Clinical evidence has supported aggressive treatment for oligometastases. Tomlinson et al. analyzed the survival of patients with colorectal liver metastases who underwent lesion resection of the liver metastasis and found out that10-year overall survival was far better than those of patients treated with systemic therapy [14]. Liver metastasectomy of breast cancer, neuroendocrine tumors and melanoma has also shown benefit for the patients and prolonged patients' survival [15-17]. Other studies have shown better prognosis of oligometastatic cancer patients who had been treated with stereotactic body radiation (SBRT) [18, 19]. SBRT has also shown benefits for patients with oligometastatic bone metastasis for prolonged survival, pain relief and safety [20]. Also current studies have indicated that prostate cancer may be amenable to more aggressive local ablative therapy with prolonged local control and delay to androgen deprivation therapy [21].

BMs are frequently present in patients with mRCC causing significant morbidity. Recent evidences suggest that BMs are associated with poor clinical outcomes in patients with mRCC. McKay et al. analyzed 2749 patients from 2003 to 2011 in phase 2 and 3 trials and found the presence of BMs in patients was correlated with shorter overall survival (OS) if compared with patients without BMs (13.2 vs 20.2 months, p<0.0001) [22]. One study that involved patients under the treatment of sunitinib shared the same point of view. Overall survival (OS) was significantly shorter in patients with bone metastases than in those without BMs (19.5 vs 38.5 months, P<0.0001) [23].

However, in our study, we revealed that RCC patients with less than 5 sites of bone metastasis have a favorable outcome under the treatment of sunitinib. Long-term survival is expected for oligometastaic patients (30.1 months vs 12.7 months). The incorporation of metastatic state into one of the established prognostic models (the MSKCC risk model) improved its predictive accuracy. Therefore, oligometastaic state is a promising prognostic factor of mRCC if treated with sunitinib.

Also, our study identified other prognostic factors affecting the outcome of patients with bone metastasis. We showed that ECOG-PS, MSKCC score and LDH level were associated with OS. Moreover, the presence of lymph-node metastasis was independent prognostic factors in patients with BMs.

The major strength of this study is that it is a clinical review with a long-term follow-up (form 2008 to 2015). All the patients were under the treatment of sunitinib after nephrectomy (Most of them were treated as first-line therapy. Only 3 of them used sunitinib after the use of sorafenib), which avoided the bias of the treatment option. There are certain limitations. Our study only included patients in a single center with limited population. Plus, it was a retrospective study. We didn't treat oligometastatic patients with further aggressive measures like lesion resection or SBRT. Further prospective studies with multi-centers and large population are urged.

## CONCLUSION

RCC patients with oligometastatic state of bone metastasis treated with sunitinib had a favorable clinical outcome. Further multi-center with larger population and prospective studies are urged.
